# Mechanisms and Characterization of *Trichoderma longibrachiatum* T6 in Suppressing Nematodes (*Heterodera avenae*) in Wheat

**DOI:** 10.3389/fpls.2017.01491

**Published:** 2017-09-15

**Authors:** Shuwu Zhang, Yantai Gan, Weihong Ji, Bingliang Xu, Baohong Hou, Jia Liu

**Affiliations:** ^1^Biocontrol Engineering Laboratory of Crop Diseases and Pests of Gansu Province, College of Plant Protection, Gansu Agricultural University Lanzhou, China; ^2^Gansu Provincial Key Laboratory of Arid Land Crop Science, Gansu Agricultural University Lanzhou, China; ^3^Swift Current Research & Development Centre, Agriculture and Agri-Food Canada Swift Current, SK, Canada; ^4^Human-Wildlife Interactions Research Group, Institute of Mathematical and Natural Sciences, Massey University Auckland, New Zealand

**Keywords:** *Trichoderma* spp., *Heterodera avenae*, eggs and second stage juveniles, parasitic and lethal effects, biological control, antioxidative defense system

## Abstract

*Heterodera avenae* is an important soil-borne pathogen that affects field crops worldwide. Chemical nematicides can be used to control the nematode, but they bring toxicity to the environment and human. *Trichoderma longibrachiatum* has been shown to have the ability to control *H. avenae* cysts, but detailed microscopic observations and bioassays are lacking. In this study, we used microscopic observations and bioassays to study the effect of *T. longibrachiatum* T6 (TL6) on the eggs and second stage juveniles (J2s) of *H. avenae*, and investigate the role of TL6 in inducing the resistance to *H. avenae* in wheat seedling at physiological and biochemical levels. Microscopic observations recorded that TL6 parasitized on the *H. avenae* eggs, germinated, and produced a large number of hyphae on the eggs surface at the initial stage, thereafter, the eggs were completely surrounded by dense mycelia and the contents of eggs were lysed at the late stage. Meanwhile, the conidia suspension of TL6 parasitized on the surface of J2s, produced a large number of hyphae that penetrated the cuticle and caused deformation of the nematodes. TL6 at the concentration of 1.5 × 10^7^ conidia ml^−1^ had the highest rates of parasitism on eggs and J2s, reflected by the highest hatching-inhibition of eggs and the mortality of J2s. In the greenhouse experiments, wheat seedlings treated with TL6 at 1.5 × 10^7^ conidia ml^−1^ had reduced *H. avenae* infection, and increased plant growth significantly compared to the control. The cysts and juveniles in soil were reduced by 89.8 and 92.7%, the juveniles and females in roots were reduced by 88.3 and 91.3%, whereas the activity of chitinase and β-1, 3-glucanase, total flavonoids and lignin contents in wheat roots were increased significantly at different stage after inoculation with the eggs and TL6 conidia in comparison to the control. Maximum activity of chitinase and β-1, 3-glucanase were recorded at the 20^th^ and 15^th^ Days after inoculation with TL6 and thereafter it declined. The maximum contents of total flavonoids and lignin were recorded at the 35^th^ and 40^th^ Days after inoculation with TL6. After being stained with the rapid vital dyes of acridine orange (AO) and neutral red (NR), the frozen and infected eggs and J2s of *H. avenae* changed color to orange and red, respectively, while the color of eggs and J2s in control group did not change. Therefore, our results suggest that TL6 is potentially an effective bio-control agent for *H. avenae*. The possible mechanisms by which TL6 suppresses *H. avenae* infection are due to the direct parasitic and lethal effect of TL6 on the eggs and J2s activity, and the induced defense response in wheat plants together.

## Introduction

Plant parasitic nematodes are one of the most important pathogens causing plant diseases, affecting the growth, yield and quality of crops, which results in economic losses (Bird and Kaloshian, 2003; Wei et al., 2014). It is estimated that the annual cost due to parasitic nematodes is about US $157 billion in crop production worldwide (Abad et al., 2008). Among pathogenic nematodes, *Heterodera glycines, Heterodera avenae, Heterodera schachtii, Globodera rostochiensis, Globodera pallida*, and *Meloidogyne* spp. are most economically important (Barker and Noe, 1987; Nicol and Rivoal, 2008). *H. avenae* causes “Molya” disease in wheat (*Triticum aestivum* L.) and barley (*Hordeum vulgare* L.). In India, *H. avenae* infection causes yield loss in wheat by 47.2% and in barley by 87.2% (Rivoal and Cook, 1993). In China, with the increase of wheat growing areas, the impact of *H. avenae* has become a serious concern and innovative management measures are required to combat the disease (Nicol et al., 2007; Li et al., 2010). Since first reported in Hubei in 1989, *H. avenae* is now widely distributed and has been recorded in Hebei, Henan, Beijing, Shanxi and other provinces. It is estimated that more than one million hectares of wheat is affected each year, with the yield loss up to 50% (Peng et al., 2009; Riley et al., 2010; Yuan et al., 2010). In cereal crops, *H. avenae* can feed on the crop roots and cause root wound, providing opportunity for *Rhizoctonia solani* infection and seedling rot (Peng et al., 2009).

Chemical and biological nematicides are currently used to control nematode infection in agricultural crops (El-Alfy and Schlenk, 2002; Huang et al., 2014). For example, the nematicides Carbofuran, Etho-prophos, and Miral have about 90% efficacy in controlling of *H. avenae*, but these nematicides are toxic to the beneficial microorganisms in soil (Sergio, 2011). Moreover, some of the nematicides are difficult to degrade in soil which can cause pollution of underground water and the environment (Jatala, 1986). Some synthetic nematicidal agents are not species specific and can produce toxins that kill other symbiotic organisms in rhizosphere (Sergio, 2011). Because of the limitations of chemical control, biological control has been considered as an alternative. A great number of potential biological control microorganisms have been isolated, including fungi (predatory fungus, wireworm parasitic fungus, egg parasitic fungus, poisonous fungus and mycorrhizal fungus) (Crump et al., 1983; Sergio, 2011), bacteria (Chen and Dickson, 1996), and protozoon (Becker et al., 1988). Some biological control microorganisms such as *Paecilomyces lilacinus* and *Pochonia chlamydosporia* have been tested under field conditions (Sun et al., 2006). However, the effectiveness of these microbial agents is limited, as the microorganism *P. lilacinus* usually parasitizes nematode eggs and the rate of infection is related to the duration of the infection (Leij De et al., 1992; Bonants et al., 1995). *Pochonia chlamydosporia* usually infects eggs and females and the ability to colonize plant rhizosphere differs with plant species (Oclarit and Cumagun, 2009), thus, it is ineffective in parasitizing the eggs of root nematodes (Sun et al., 2006). It is imperative to identify and develop effective bio-control agents for preventing and controlling plant parasitic nematodes.

*Trichoderma* spp. is one of the important groups of rhizosphere fungi widely distributed in soil. Some species, such as *T. hatzianum* and *T. viride* have been proven to be good antagonists against some soil-borne plant pathogens such as *Rhizoctonia* spp., *Sclerotium* spp., *Fusarium* spp., and *Pythium* spp. (Bourne et al., 1996; Samuels, 1996; Deng et al., 2007). *Trichoderma* formulations have been commercialized in some countries, i.e., *T. harzianum* T22 (Topshield) in the United States (Louzada et al., 2009) and the strain of *T. harzianum* T39 (Trichodex) in Israel (Elad, 2000). In our previous studies, we found that the strain of *T. longibrachiatum* T6 (TL6) has a remarkable effect on alleviating the adverse effects of abiotic stress on wheat seedling growth and development (Zhang et al., 2016). Further, we found the fermentation broth of *T. longibrachiatum* has a great potential to be used as a bio-control agent against *H. avenae* cysts (Zhang et al., 2014a,b). The spore suspension of *T. longibrachiatum* was found highly effective against *M. incognita* (Zhang et al., 2015). However, the process of the conidia suspension of TL6 in controlling *H. avenae* eggs and second stage juveniles (J2s) has not been reported. Little information is available in regard to the effectiveness of TL6 in controlling nematodes, especially on the parasitic and inhibitory effects on the eggs and J2s of *H. avenae*. In the present study, we (i) assessed the infection process of TL6 on eggs and J2s of *H. avenae* and (ii) determined the mechanisms and the effectiveness of the conidia suspension of TL6 in the control of *H. avenae* by greenhouse experiments and in vitro tests at physiological and biochemical levels.

## Materials and methods

Experiments were carried out at the Laboratory of Plant Pathology, College of Plant Protection, Gansu Agricultural University. The soil samples were collected from a wheat field in Xingyang, Henan province, China. *H. avenae* cysts were isolated from the soil and obtained using the “Flotation separation” method, and sterilized with 1% NaOCl for 1 min, and then were gently washed six times with sterile water to remove NaOCl (Long et al., 2012). The cysts were then crushed to obtain eggs using a tissue grinder. The surface of eggs was sterilized with 1% NaOCl for 30 s and washed six times with sterile water. The final concentration of the eggs was prepared to 2 ± 1 per 10 μl and 100 ± 5 per 50 μl of sterile water. For obtaining the fresh hatched J2s, the sterilized eggs were collected on the size of 25 μm mesh sieve and transferred to the Petri dishes containing sterilized tap water. The hatched fresh J2s of *H. avenae* were collected every day by incubating eggs in extraction Petri dishes at 20°C for 14 Days, and stored in sterilized tap water at 4°C. The concentration of the J2s for inoculation was then prepared to 2 ± 1 per 10 μl and 100 ± 5 per 50 μl of sterile water.

### Fungal inoculum preparation

*Trichoderma longibrachiatum* T6 (TL6) was isolated from a rhizisphere soil of a forest site nearby Tianshui, Gansu. Some basic tests were conducted at the Plant Pathology Laboratory of Gansu Agricultural University and it was reported that the strain has no hazardous effects to the environments (Zhuang et al., 2006). The strain of TL6 has since been collected at the China General Microbiological Culture Collection Center, in Beijing, with the patent number (CGMCC No.13183). For the present experiments, the strain of TL6 was cultured on potato dextrose agar (PDA) in Petri dishes for 6 Days at 25°C. The conidia suspension of TL6 was prepared according to the methods described by Zhang et al. (2014b). Final conidia suspension of different densities, 1.5 × 10^7^, 1.5 × 10^6^, 7.5 × 10^5^, 3.0 × 10^5^, and 1.5 × 10^5^ of TL6 conidia per ml, were prepared and stored at 4°C.

*Fusarium oxysporum* (FO) was obtained from the Plant Pathology Laboratory, Gansu Agricultural University and cultured on potato dextrose agar (PDA) medium at 25°C for 7 Days, and filtered into sterilized beakers. The spore concentration was determined using a hemacytometer, and diluted with sterile water to a final concentration of 1.5 × 10^7^ conidia per ml.

### Effects of different concentrations of TL6 on egg hatching of *H. avenae*

This experiment was replicated three times. The treatments included five different concentrations of TL6 (1.5 × 10^7^, 1.5 × 10^6^, 7.5 × 10^5^, 3.0 × 10^5^, and 1.5 × 10^5^ conidia ml^−1^) and the two controls with one treated with sterile water and the other treated with *F. oxysporum* suspension. For each treatment and each replicate, surface sterilized eggs of 100 ± 5 suspended in sterile water were placed in each Petri dish, and counted under a stereomicroscope. After confirmation of the number of eggs in each plate, 5 ml of TL6 suspension were added to the plates for each of the five TL6 concentration treatments, whereas 5 ml of sterile water or *F. oxysporum* suspension were added to the control plates. Hatching (number of J2s emerged from eggs) and parasitism were recorded on the 8^th^ Day after the treatment. Percentages of inhibition and parasitism were calculated according to Gao et al. (1998).

(1) $$\begin{array}{r} \text{PPE }\left(\%\right)=\left(\text{NEPET}/\text{TNTE}\right)\;\times \;100 \end{array}$$

Where PPE represents percentages of parasitism on eggs, NEPET number of eggs parasitized in each treatment and TNTE the total number of test eggs.

(2) $$\begin{array}{r} \text{RPIEH }\left(\%\right)=\left(\text{NJHSWCG}-\text{NJHET}\right)/\text{NJHSWCG }\times \;100 \end{array}$$

Where RPIEH represents relative percentages of inhibition of eggs hatching, NJHSWCG number of J2s hatched in sterile water control group and NJHET number of J2s hatched in each treatment.

### Effects of different concentrations of TL6 on J2s of *H. avenae*

This experiment was replicated three times. The treatments included five different concentrations of TL6 and the two controls with one treated with sterile water and the other treated with *F. oxysporum* suspension. For each treatment and each replicate, surface sterilized J2s of 100 ± 5 suspended in sterile water were placed in each 6-well sterilized cell culture plate, and counted under a stereomicroscope. After confirmation of the number of J2s in each plate, 3 ml of TL6 suspension were added to each well for each of the five TL6 concentration treatments, whereas 3 ml of sterile water or *F. oxysporum* suspension were added to the control plates.

After 24, 48, and 72 h of treatment, the remaining J2s were prodded with a needle and those that did not respond were considered dead. The percentages of immotile nematodes were calculated (Meyer et al., 2004). Linear regression was used to evaluate the effect of different concentrations of TL6 on the corrected motility rate of J2s of *H. avenae* (Khan et al., 2006). Mortality and the corrected mortality of *H. avenae* J2s were calculated using the equations described by Zhang et al. (2015).

(3) $$\begin{array}{r} \text{M }\left(\%\right)\;=\;\left(\text{NDJET}/\text{TNTJET}\right)\;\times \;100 \end{array}$$

Where M represents mortality, NDJET number of dead J2s in each treatment and TNTJET the total number of test J2s in each treatment.

(4) $$\begin{array}{r} \text{CM }\left(\%\right)\;=\;\left(\text{MET}-\text{MSWCG}\right)/\left(1-\text{MSWCG}\right)\;\times 100 \end{array}$$

Where CM represents corrected mortality, MET the mortality in each treatment and MSWCG the mortality in sterile water control group.

The parasitic effect of different concentrations of TL6 on the J2s of *H. avenae* was observed every 2 Days after the treatment from the 6^th^ to 14^th^ Day, and percentages of parasitism were calculated using the equation of Zhang et al. (2015).

(5) $$\begin{array}{r} \text{PPSSJ }\left(\%\right)\;=\;\left(\text{NSSJPET}/\text{TNTSSJ}\right)\;\times \;100 \end{array}$$

Where PPSSJ represents percentages of parasitism on second stage juveniles, NSSJPET number of second stage juveniles parasitized in each treatment and TNTSSJ the total number of test second stage juveniles.

### Microscopic observation of the infection process of TL6 on eggs and J2s of *H. avenae*

This experiment was replicated six times with the seven treatments the same as those described above. For each treatment and each replicate, 10 μl (2 ± 1 eggs) of surface sterilized eggs suspended in sterile water were placed in each sterilized Petri dish of 3.5 cm in diameter. After that the suspension of TL6 (990 μl) was added to each sterilized Petri dish of 3.5 cm in diameter for each of the five TL6 concentration treatments, whereas sterile water or *F. oxysporum* suspension (990 μl) was added to the two separate control plates. The process of infection was observed using a stereomicroscope. The daily observations started on the 2nd Day after the treatment until the 12^th^ Day. In each day of the observation, the detailed information was recorded for the same eggs.

Following the same protocol of the observation on eggs, we observed the infection of TL6 on J2s for each of the seven treatments and each of the six replicates. Ten μl of suspension containing 2 or 3 J2s were pipetted into each sterilized Petri dish of 3.5 cm in diameter, and TL6 suspension (990 μl) was then added to each culture plate for each of the five TL6 concentration treatments, whereas the same amount of sterile water or *F. oxysporum* suspension was used in the two controls, respectively. The daily observations started on the 2nd Day after the treatment until the 12^th^ Day. In each day of the observation, the detailed information was recorded for the same J2s.

### A rapid method for assaying the viability of nematodes

Two vital dyes were used to determine the viability of *H. avenae* eggs and J2s *in vitro*. Acridine orange (AO, Sigma A6014, US) and neutral red (NR, Solarbio-N8160, Amresco-E89) were dissolved in sterilized distilled water and the dyeing was added to the incubation solution to make (i) 0.01% concentration of AO and (ii) 0.01% concentration of NR. This experiment included two treatments and replicated six times. The first treatment was under ultra-low temperature freezer (−80°C) for 15 min, and the second treatment was with the suspension (1.5 × 10^7^ conidia ml^−1^) of TL6. The detailed treatment procedures are as follow:

For the first treatment, the suspensions of surface sterilized eggs or J2s (2 ± 1 per 10 μl) in 1.5 ml centrifuge tubes were placed in ultra-low temperature freezer (−80°C) for 15 min, and then moved to a water bath (60°C) for 1 min to thaw quickly. The surface sterilized eggs with the similar stage of growth or J2s were maintained at 4°C as the control. Also, 10 μl of eggs or J2s in each treatment were placed into each well of sterilized cell culture plate (96 well) in the treatment group, and 90 μl of the dye solutions were added to each 96 well cell culture plate, and mixed thoroughly until the dyes fully diffused. The eggs were stained for 15 min whereas J2s were stained for 30 min before being rinsed with sterilized distilled water. The viability of eggs and J2s was identified using an optical microscope.

For the second treatment, the surface sterilized eggs or J2s (2 ± 1 per 10 μl) were placed into each 96 well sterilized cell culture plate, and then the suspension (1.5 × 10^7^ conidia ml^−1^) of TL6 (90 μl) was added to each plate in the treatment group and sterilized water (90 μl) was added to the control. The eggs and J2s in the treatment and the control group were stained at Day 8 of incubation treatment. The dye solution of 100 μl was added to each 96 well sterilized cell culture plate, and mixed thoroughly until the dyes fully diffused. The eggs were stained for 15 min whereas J2s were stained for 30 min before being rinsed with sterilized distilled water. The viability of eggs and J2s was identified using an optical microscope.

### Bio-control experiment in greenhouse

This experiment included seven treatments and replicated three times. The wheat cultivar Yongliang 4, susceptible to *H. avenae*, was used in the experiment. Wheat seeds were surface sterilized with 1% sodium hypochlorite (NaOCl, 5 min) and sown in 15 cm diameter pots containing 500 g sterile soil (silty clay: sand = 3:1 v/v). Ten seedlings per pot were grown in a greenhouse with air temperature of 25°C ± 0.5, and supplemental day/night lighting of 16/8 h. Pots were irrigated daily with sterilized distilled water which enabled the relative humidity to be maintained around 65%. The experiment was arranged using a completely randomized design. Each replicate had 18 pots, allowing multiple sampling (described below). When seedlings reached 10 cm in height, about 15 Days after sowing, each pot was inoculated with 1,500 ± 100 eggs of *H. avenae*. Ten Days after the inoculation, seedlings were inoculated with 20 ml of TL6 conidia suspension. The treatments included the five concentrations (1.5 × 10^7^, 1.5 × 10^6^, 7.5 × 10^5^, 3.0 × 10^5^, and 1.5 × 10^5^ conidia ml^−1^) of TL6 conidia and the two controls (i) seedlings inoculated with eggs but not with TL6 (replaced with sterilized distilled water), and (ii) seedlings not inoculated with eggs or TL6 (Mock-inoculated plants (normal)).

The fresh roots of wheat seedlings were sampled six times at 5 or 10 days intervals (i.e., 5, 10, 15, 20, 30, and 40 Days) after inoculation with eggs and TL6 conidia suspension. Chitinase and β-1, 3-glucanase activity, the contents of lignin and total flavonoids were assayed:

#### Chitinase and β-1, 3-glucanase activity

For the determination of enzyme activity, the enzyme extracts of the fresh roots were prepared following the methods of El Ghaouth et al. (2003) with some modifications. Fresh roots of 1 g were homogenized in 5 ml of buffer that contained 50 mM sodium acetate (pH 5.0), and then the homogenate was filtered and centrifuged at 10,000 g for 30 min. The supernatant was retained and used as the enzyme extract to assay the chitinase and β-1, 3-glucanase activity. The experiments were repeated six times.

Chitinase and β-1, 3-glucanase activity were assayed according to the method of El-Katatny et al. (2001). For the activity of chitinase, 50 mM sodium acetate buffer (pH 5.0) and 0.2 ml of 0.5% colloidal chitin were added to 0.5 ml of the supernatant of the wheat seedling enzyme extracts. The activity of chitinase was measured by assaying the quantity of reducing sugar as described by Adney and Baker (2008). The absorbance of the reaction mixture was determined at 585 nm using the spectrophotometer (UV), and the quantities of reducing sugar were calculated from a calibration curve with N-acetyl glucosamine (N-AcG) as the standard. The activity of chitinase was expressed as nmol N-AcG mg^−1^ protein min^−1^.

Activity of β-1, 3-glucanase (expressed as nmol glucose mg^−1^ protein min^−1^) was determined and performed by using 5% laminarin as a substrate. The reaction mixture was mixed with 0.5 ml of extract, 50 mM sodium acetate buffer (pH 5.0) and 0.2 ml of 5% laminarin, and thereafter was incubated in a water bath at 37°C for 20 min. The activity of β-1, 3-glucanase was determined by assaying the quantity of reducing sugar in the reaction mixture. The absorbance was read at 540 nm by a colorimetric assay, and a calibration curve was prepared by using glucose as the standard (El-Katatny et al., 2001).

#### Lignin and total flavonoids

The contents of lignin in wheat seedling roots were measured following the method of Lee et al. (2007), with minor modifications. The dry weight of 0.5 g root samples were homogenized with distilled water and 95% ethanol twice to remove the metabolites of soluble sugar. Thereafter, the supernatant of extraction was discarded after being centrifuged at 10,000 g for 5 min, and the insoluble residue was left to dry at 45°C overnight. The dried samples were washed with 1 ml of acetyl bromide with acetic acid (1:3, v/v), and then left at 70°C for 30 min. After that, the supernatant was discarded and the precipitate was re-suspended in 0.36 ml of NaOH (2 M) and 0.04 ml of hydroxylamine hydrochloride (7.5 M), and then acetic acid was added to the mixture to make up the final volume to 10 ml when it cooled down to room temperature. The absorbance of the supernatant was measured at 280 nm after the final reaction mixture was centrifuged at 1,000 g for 5 min (Lin and Kao, 2001), and the contents of lignin were calculated according to the linear calibration curve with lignin as the standard (Lee et al., 2007). All treatments were repeated six times.

The reaction mixtures of wheat seedling fresh roots were extracted following the procedure of Hertog et al. (1992). The contents of total flavonoids were measured as gallic acid using Folin Ciacalteau reagent (Ragazz and Veronese, 1973) with a modification. Fresh roots samples of 1 g were crushed to 5 ml of 1% hydrochloric acid-methanol for 24 h, and then the extracts were diluted to 25% of original concentration. After that, the diluted extracts (0.2 ml) were mixed with 0.5 ml of Folin Ciacalteau reagent and 5 ml of distilled water. After a 5 min reaction, the reaction mixture was neutralized with 3 ml of 20% Na_2_CO_3_, vortexed and left for 30 min at room temperature. The absorbance was measured at 725 nm using a spectrophotometer. The total flavonoids were expressed as mg of gallic acid per g of wheat seedling fresh roots. The experiment was repeated six times.

#### Growth traits of wheat seedlings and nematode populations in the rhizosphere

Sixty-five Days after sowing, plant height, root length, shoot and root fresh weights of wheat seedlings inoculated with eggs and the different concentrations of TL6 were measured. The number of cysts, females and juveniles were recorded and assessed in both soil and roots after inoculation with eggs and different concentrations of TL6. *Heterodera avenae* cysts in the soil were extracted from 200 g of soil samples per pot using “Flotation separation” method (Long et al., 2012), and *H. avenae* juveniles in the soil were extracted from 20 g of soil samples per pot using centrifugation technique (Castillo et al., 2006). Nematode root densities were assessed from 2 g of root sub-samples of each plant (Sharon et al., 2007).

### Statistical analysis

In the study, replicated observations were made randomly and independently of each other and they were in a normal distribution with common variances, thus, the ANOVA assumption was generally met. Our treatments was essentially one factor only, therefore, one-way ANOVA was performed to determine the treatment effect using SPSS Version 16.0 (SPSS Inc., Chicago, IL). The significant differences between the treatments were considered at the level of *P* < 0.05. Fisher's least significant difference (LSD) values were computed using standard error and *T*-values of adjusted degrees of freedom. Linear regression was used to determine the relationship between the corrected mortality (%) and the corresponding values of the concentrations of the conidia suspension of TL6. The percentage values were log-transformed prior to statistical analysis.

## Results

### Microscopic observation of the infection process of TL6 on *H. avenae* eggs

**Figure 1:**
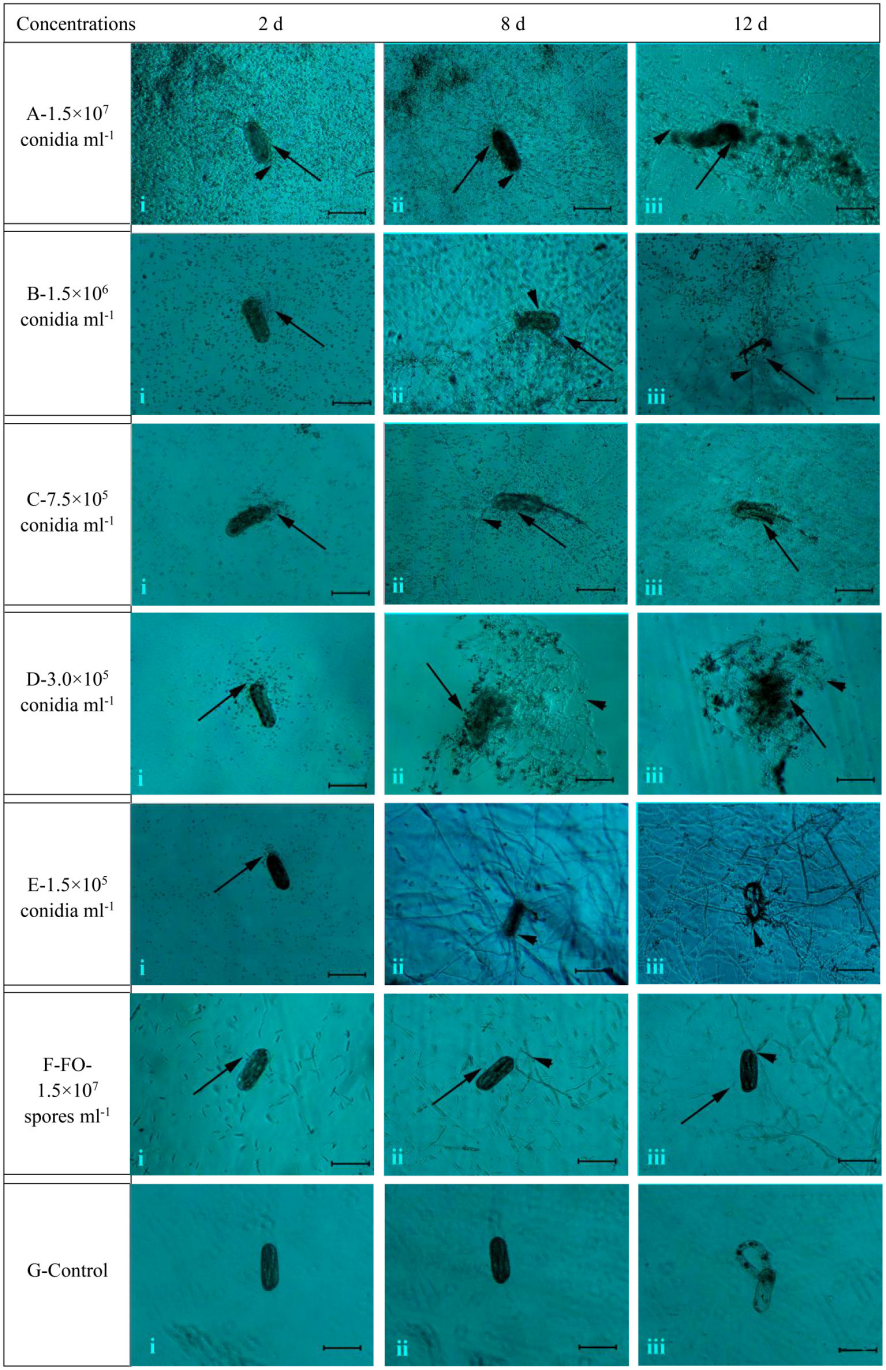
The morphological characteristics of the eggs of *H. avenae* were infected by the conidia suspension of TL6 *in vitro*. Observations were made at **(i)** 2 Days, **(ii)** 8 Days, and **(iii)** 12 Days after treatment with TL6 conidia suspension. Bars are in the unit of 100 μm in all the cases. 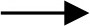 represents spores; 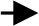 represents mycelia.

Overall, the different concentrations of conidia suspension of TL6 (1.5 × 10^7^, 1.5 × 10^6^, 7.5 × 10^5^, 3.0 × 10^5^, and 1.5 × 10^5^ conidia ml^−1^) had the different inhibitory and parasitic effects on the hatching of *H. avenae* eggs, with higher concentrations of TL6 presenting a stronger and more significant inhibitory and parasitic effects (Figure 1). At the concentration of 1.5 × 10^7^ conidia ml^−1^, a large number of conidia of TL6 adhered and surrounded the surface of eggs at the early stages (at Day 2) of infection (Figure 1Ai), and the conidia germinated and produced a large number of hyphae parasitized on the surface at Day 8 (Figure 1Aii). The content of eggs was dissolved by the metabolites of TL6 at Day 12 (Figure 1Aiii). At the concentration of 1.5 × 10^6^ conidia ml^−1^, many conidia adhered to the surface of eggs at Day 2 (Figure 1Bi). With the increase of incubation time, the conidia parasitized and grew on the surface of eggs. Fewer conidia germinated hyphae and grew on the eggs at Day 8 (Figure 1Bii). The germinated hyphae penetrated into the eggs shell and dissolved the content and eggs shell at Day 12 (Figure 1Biii). As the TL6 concentration was reduced to 7.5 × 10^5^ conidia ml^−1^, only a few conidia adhered and parasitized on the surface of eggs at Day 2 of inoculation (Figure 1Ci). The hatched nematodes body and the eggs shell were parasitized with the hyphae at Day 8 (Figure 1Cii), and were dissolved at Day 12 (Figure 1Ciii). With the TL6 concentrations were reduced further to 3.0 × 10^5^ and 1.5 × 10^5^ conidia ml^−1^, only a few conidia surrounded the eggs at Day 2 after inoculation (Figures 1Di,Ei). However, the eggs were parasitized with the conidia and dense mycelium at 3.0 × 10^5^ conidia ml^−1^ at Day 8 (Figure 1Dii), while the dense mycelium parasitized, penetrated and grew on the eggs at 1.5 × 10^5^ conidia ml^−1^ at Day 8 (Figure 1Eii). The content and shell of eggs were completely dissolved at the concentration of 3.0 × 10^5^ conidia ml^−1^ of treatment at Day 12 (Figure 1Diii), whereas the parasitized dense mycelium was grown and penetrated into the eggs shell, and the contents of eggs were dissolved after inoculated with the concentration of 1.5 × 10^5^ conidia ml^−1^ at Day 12 (Figure 1Eiii). In contrast, the strain of *F. oxysporum* in the control had a lower inhibitory and parasitic effect on the hatching of *H. avenae* eggs. Some conidia surrounded the eggs at Days 2 and 8 (Figures 1Fi,ii), but only a few of them geminated hyphae and grew on the surface of eggs at Day 12 (Figure 1Fiii). No inhibitory and parasitic effects were found in the sterile water control. No mycelium was formed and the embryonic development was normal in the sterile water control group (Figures 1Gi,ii) and also nematodes begun to hatch at Day 12 (Figure 1Giii).

### Effects of different concentrations of TL6 on *H. avenae* eggs

With the increase of TL6 concentrations (1.5 × 10^7^, 1.5 × 10^6^, 7.5 × 10^5^, 3.0 × 10^5^, and 1.5 × 10^5^ conidia ml^−1^), the inhibitory and parasitic effects on the *H. avenae* eggs hatching rates were increased, while the strain of *F. oxysporum* in the control had a lower inhibitory and parasitic effect, and the sterile water control had no inhibitory and parasitic effect (Tables 1, 2).

**Table 1:** Effects of different concentrations of TL6 on parasitism of *H. avenae* eggs.

**Concentrations (conidia ml^−1^)**	**Days after incubation (d)**				
	**8**	**9**	**10**	**11**	**12**
	**Percent parasitism (%)**				
1.5 × 10^7^	70.7 ± 2.2a	77.7 ± 4.9a	87.7 ± 2.4a	90.3 ± 1.5a	91.3 ± 0.9a
1.5 × 10^6^	63.7 ± 4.1a	77.3 ± 2.0a	84.7 ± 3.0a	87.3 ± 2.2a	90.3 ± 2.0a
7.5 × 10^5^	48.7 ± 3.2b	56.7 ± 2.6b	64.7 ± 3.5b	71.3 ± 3.2b	79.3 ± 2.6b
3.0 × 10^5^	39.7 ± 4.3c	43.0 ± 3.1c	50.7 ± 3.5c	60.0 ± 2.1c	68.7 ± 1.9c
1.5 × 10^5^	16.3 ± 2.3d	23.7 ± 4.2d	27.7 ± 3.8d	38.0 ± 3.8d	40.7 ± 2.6d
FO-1.5 × 10^7^	15.3 ± 0.9d	21.7 ± 1.5d	29.0 ± 1.2d	31.7 ± 1.3d	36.3 ± 0.9e
Control	0.0 ± 0.0e	0.0 ± 0.0e	0.0 ± 0.0e	0.0 ± 0.0e	0.0 ± 0.0f
Mean square	2,125.11	2,622.54	3,135.65	3,219.19	3,429.64
*F* value	89.08	95.78	134.11	200.60	344.06
*P* value	0.0001	0.0001	0.0001	0.0001	0.0001

*Data are means ± SE in Experiment. More than three hundreds surface sterilized eggs were tested in each treatment. Values in columns followed by different letters are significantly different at P < 0.05 based on Fisher's LSD test. FO-1.5 × 10^7^ represents eggs treated with the spore suspension of F. oxysporum at the concentration of 1.5 × 10^7^ spores ml^−1^. Control represents eggs treated with sterile water*.

**Table 2:** Effects of different concentrations of TL6 on relative percentages of hatching inhibition of *H. avenae* eggs.

**Concentrations (conidia ml^−1^)**	**Days after incubation (d)**				
	**8**	**9**	**10**	**11**	**12**
	**Relative percentages of inhibition (%)**				
1.5 × 10^7^	100.0 ± 0.0a	99.2 ± 0.8a	92.1 ± 3.3a	88.4 ± 1.8a	88.4 ± 0.5a
1.5 × 10^6^	100.0 ± 0.0a	95.4 ± 4.6ab	91.5 ± 3.3a	84.6 ± 1.5a	85.0 ± 0.4b
7.5 × 10^5^	97.4 ± 1.3a	86.8 ± 4.8bc	75.0 ± 0.8b	67.4 ± 1.2b	66.9 ± 1.8c
3.0 × 10^5^	89.7 ± 1.1a	77.5 ± 2.3c	68.3 ± 0.7c	63.5 ± 0.9b	60.2 ± 1.5d
1.5 × 10^5^	69.2 ± 1.4b	63.6 ± 2.8d	48.8 ± 1.0d	55.4 ± 0.9c	49.3 ± 2.8e
FO-1.5 × 10^7^	32.1 ± 4.0c	28.7 ± 5.0e	30.5 ± 1.4e	29.6 ± 1.2d	28.2 ± 1.1f
Control	–	–	–	–	–
Mean square	2,150.32	2,041.13	1,787.40	1,368.90	1,529.99
*F* value	207.98	49.73	139.11	274.36	203.53
*P* value	0.0001	0.0001	0.0001	0.0001	0.0001

*Data are means ± SE in Experiment. More than three hundreds surface sterilized eggs were tested in each treatment. Values in columns followed by different letters are significantly different at P < 0.05 based on Fisher's LSD test. FO-1.5 × 10^7^ represents eggs treated with the spore suspension of F. oxysporum at the concentration of 1.5 × 10^7^ spores ml^−1^. Control represents eggs treated with sterile water*.

There was a trend that the number of eggs parasitized increased with the increase of TL6 concentrations, and the two highest concentrations of 1.5 × 10^7^ and 1.5 × 10^6^ conidia ml^−1^ resulted in highest percent parasitism (*P* < 0.01; Table 1). This effect increased with the increase of the treatment days. In contrast, no fungi parasitized the eggs in the sterile water control. *In vitro*, with the increase of the TL6 concentrations, the relative percentages of inhibition of eggs hatching were increased (Table 2). The relative percentages of eggs hatching-inhibition were highest at Day 8 (100.0%) and Day 9 (99.2%) and the effect decreased with the treatment days (*P* < 0.01). Among the five concentrations of TL6, the highest percentages of egg hatching inhibition were obtained with the highest concentrations at 1.5 × 10^7^ conidia ml^−1^ (*P* < 0.01).

### Microscopic observation of the infection process of TL6 on J2s of *H. avenae*

**Figure 2:**
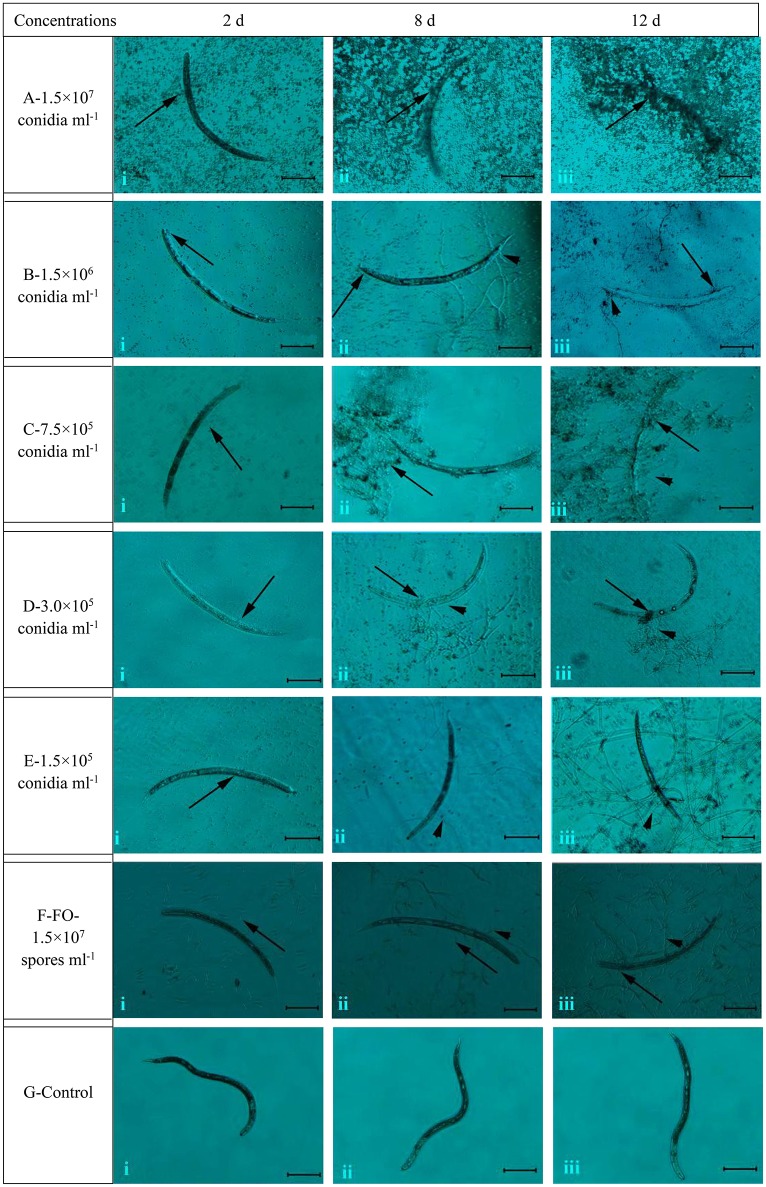
The morphological characteristics of the J2s of *H. avenae* were infected by the conidia suspension of TL6 *in vitro*. Observations were made at **(i)** 2 Days, **(ii)** 8 Days, and **(iii)** 12 Days after treatment with TL6 conidia suspension. Bars are in the unit of 100 μm in all the cases. The arrows name is detailed in the footnote of Figure 1.

The different concentrations of conidia suspension of TL6 (1.5 × 10^7^, 1.5 × 10^6^, 7.5 × 10^5^, 3.0 × 10^5^, and 1.5 × 10^5^ conidia ml^−1^) had the different parasitic and lethal effects on the J2s nematodes (Figure 2). Microscopic examination observed that when the newly hatched J2s were treated with the conidia suspension of TL6, most J2s died, and the conidia adhered at or parasitized on the surface at Day 2 at 1.5 × 10^7^ (Figure 2Ai) and 1.5 × 10^6^ conidia ml^−1^ (Figure 2Bi), whereas the J2s were treated with lower concentrations (7.5 × 10^5^, 3.0 × 10^5^, and 1.5 × 10^5^ conidia ml^−1^) became dull, stiff, and showed a wave-like distortion at Day 2, and even a small number of dead J2s were adhered or parasitized by the conidia of TL6 (Figures 2Ci,Di,Ei). At the concentration of 1.5 × 10^7^ conidia ml^−1^, a large number of conidia of TL6 surrounded or adhered to the surface of J2s at Day 8 (Figure 2Aii), and the surface of J2s was completely parasitized by the conidia of TL6 at Day 12 (Figure 2Aiii). At the concentration of 1.5 × 10^6^ conidia ml^−1^, a few conidia germinated hyphae and parasitized on the dead nematodes surface at Day 8 (Figure 2Bii), and even the dead nematodes were dissolved by the parasitized hyphae and conidia of TL6 at Day 12 (Figure 2Biii). At the concentration of 7.5 × 10^5^ conidia ml^−1^, the dead nematodes were parasitized by the conidia and hyphae of TL6 at Day 8 (Figure 2Cii). The body of J2s was completely parasitized by the dense mycelium, and even some conidia started reproduction on the parasitic sites of J2s body. The J2s began to dissolve at Day 12 (Figure 2Ciii). As the TL6 concentration was reduced to 3.0 × 10^5^ conidia ml^−1^, a few conidia parasitized on the J2s surface, and even germinated hyphae and penetrated into the J2s body at Day 8 (Figure 2Dii), while the parasitized site reproduced a few number of conidia and hyphae which grew on the surface at Day 12 (Figure 2Diii). At the concentration of 1.5 × 10^5^ conidia ml^−1^, the conidia germinated a small number of hyphae and parasitized on the J2s surface at Day 8 (Figure 2Eii). However, a large number of hyphae penetrated into the integument and the parasitic sites became shrunk by the dense mycelium at Day 12 (Figure 2Eiii). On the contrary, the strain of *F. oxysporum* had no significant inhibitory and parasitic effect on the J2s of *H. avenae*, with just a few spores surrounded the J2s at Days 2 and 8 (Figures 2Fi,ii), and only a few geminated hyphae parasitized on the surface of J2s at Day 12 (Figure 2Fiii). The activity, body′s color and shape of J2s in the control group remained intact after inoculated with the sterile water (Figures 2Gi,ii,iii).

### Effects of different concentrations of TL6 on the J2s of *H. avenae*

With the increase of TL6 concentrations (1.5 × 10^7^, 1.5 × 10^6^, 7.5 × 10^5^, 3.0 × 10^5^, and 1.5 × 10^5^ conidia ml^−1^), the percentages of parasitism on J2s of *H. avenae* increased significantly. The percentages of parasitism reached 88.7% after 14 Days of treatment with the concentration of TL6 at 1.5 × 10^7^ conidia ml^−1^ (*P* < 0.01), while the strain of *F. oxysporum* in the control had lower parasitic effect, and the sterile water control had no parasitic effect. Also, with the increase of incubation days, the percentages of parasitism on J2s of *H. avenae* increased significantly after treated with different concentrations of TL6 (Table 3).

**Table 3:** Effects of different concentrations of TL6 on parasitism of *H. avenae* J2s *in vitro*.

**Concentrations (conidia ml^−1^)**	**Days after incubation (d)**				
	**6**	**8**	**10**	**12**	**14**
	**Percent parasitism (%)**				
1.5 × 10^7^	60.3 ± 3.5a	69.0 ± 1.5a	80.3 ± 4.3a	86.7 ± 1.2a	88.7 ± 1.8a
1.5 × 10^6^	49.3 ± 2.2b	61.0 ± 2.5b	64.3 ± 3.8b	72.0 ± 1.5b	82.0 ± 3.2b
7.5 × 10^5^	42.7 ± 3.2bc	50.7 ± 2.7c	58.0 ± 2.3bc	66.0 ± 3.6bc	73.3 ± 1.5c
3.0 × 10^5^	37.7 ± 3.8cd	43.3 ± 2.2d	51.7 ± 3.0cd	61.7 ± 1.8c	67.7 ± 0.9d
1.5 × 10^5^	32.3 ± 4.1d	39.7 ± 2.8d	47.7 ± 2.8d	54.0 ± 4.0d	62.0 ± 1.5e
FO-1.5 × 10^7^	11.0 ± 1.2e	15.3 ± 0.7e	17.3 ± 0.9e	21.7 ± 1.5e	23.0 ± 2.1f
Control	0.0 ± 0.0f	0.0 ± 0.0f	0.0 ± 0.0f	0.0 ± 0.0f	0.0 ± 0.0g
Mean square	1,350.89	1,807.65	2,315.27	2,759.38	3,218.78
*F* value	52.34	143.25	96.66	167.96	326.54
*P* value	0.0001	0.0001	0.0001	0.0001	0.0001

*Data are means ± SE in Experiment. More than three hundreds J2s were tested in each treatment. Values in columns followed by different letters are significantly different at P < 0.05 based on Fisher's LSD test. FO-1.5 × 10^7^ represents J2s treated with the spore suspension of F. oxysporum at the concentration of 1.5 × 10^7^ spores ml^−1^. Control represents J2s treated with sterile water*.

Different concentrations of TL6 showed a significant lethal effect on the activity of J2s, and the highest concentration of 1.5 × 10^7^ conidia ml^−1^ resulted in highest mortality and corrected mortality (*P* < 0.01). Moreover, there was a significant linear relationship between TL6 and the J2s corrected mortality, and the increased concentrations of TL6 increased the rates of corrected mortality of J2s. The mortality or corrected mortality increased with the increase of the treatment time when treated with different concentrations of TL6. In contrast, the strain of *F. oxysporum* in the control had lower lethal effect on the activity of J2s at 1.5 × 10^7^ spores ml^−1^, and the sterile water control had no significant lethal effect on the J2s mortality regardless of the TL6 concentration (Table 4).

**Table 4:** Effects of different concentrations of TL6 on the activities of *H. avenae* J2s *in vitro*.

**Concentrations (conidia ml^−1^)**	**Times after incubation (h)**					
	**24**		**48**		**72**	
	**Mortality (%)**	**Corrected mortality (%)**	**Mortality (%)**	**Corrected mortality (%)**	**Mortality (%)**	**Corrected mortality (%)**
1.5 × 10^7^	73.7 ± 2.6a	72.5 ± 2.4a	84.0 ± 2.9a	82.6 ± 3.2a	91.3 ± 1.5a	90.4 ± 1.6a
1.5 × 10^6^	64.0 ± 3.8b	62.4 ± 4.1b	75.7 ± 1.3b	73.6 ± 1.6b	85.3 ± 1.8a	83.8 ± 1.9b
7.5 × 10^5^	59.0 ± 3.2b	57.2 ± 3.8b	69.0 ± 2.5c	66.3 ± 2.7c	78.7 ± 3.4b	76.4 ± 3.9c
3.0 × 10^5^	48.0 ± 1.7c	45.6 ± 1.2c	53.7 ± 1.2d	49.6 ± 1.1d	59.7 ± 0.7c	55.4 ± 0.9d
1.5 × 10^5^	40.3 ± 1.8d	37.6 ± 2.6c	46.3 ± 1.8e	41.7 ± 1.7e	50.3 ± 2.4d	45.0 ± 2.3e
Control	4.3 ± 1.2f	–	8.0 ± 0.6g	–	9.7 ± 0.7f	–
FO-1.5 × 10^7^	26.3 ± 1.3e	23.0 ± 2.3d	30.0 ± 1.2f	23.9 ± 0.8f	35.3 ± 2.0e	28.3 ± 2.3f
Mean square	1,705.86	968.54	2,163.49	1,436.69	2,597.05	1,763.59
*F* value	97.61	38.57	222.71	115.03	220.80	106.87
*P* value	0.0001	0.0001	0.0001	0.0001	0.0001	0.0001
Linear regression equation (y = a*x* + b)	y = 0.4499*x* + 2.4445		y = 0.5811*x* + 1.8813		y = 0.7239*x* + 1.2773	
Related coefficient (r)	0.9693		0.9593		0.9243	
LC_50_ (conidia ml^−1^)	4.79 × 10^5^		2.32 × 10^5^		5.05 × 10^4^	

*Data are means ± SE in Experiment. More than three hundreds J2s were tested in each treatment. Values in columns followed by different letters are significantly different at P < 0.05 based on Fisher's LSD test. FO-1.5 × 10^7^ represents J2s treated with the spore suspension of F. oxysporum at the concentration of 1.5 × 10^7^ spores ml^−1^. Control represents J2s treated with sterile water*.

### Viability of nematodes

**Figure 3:**
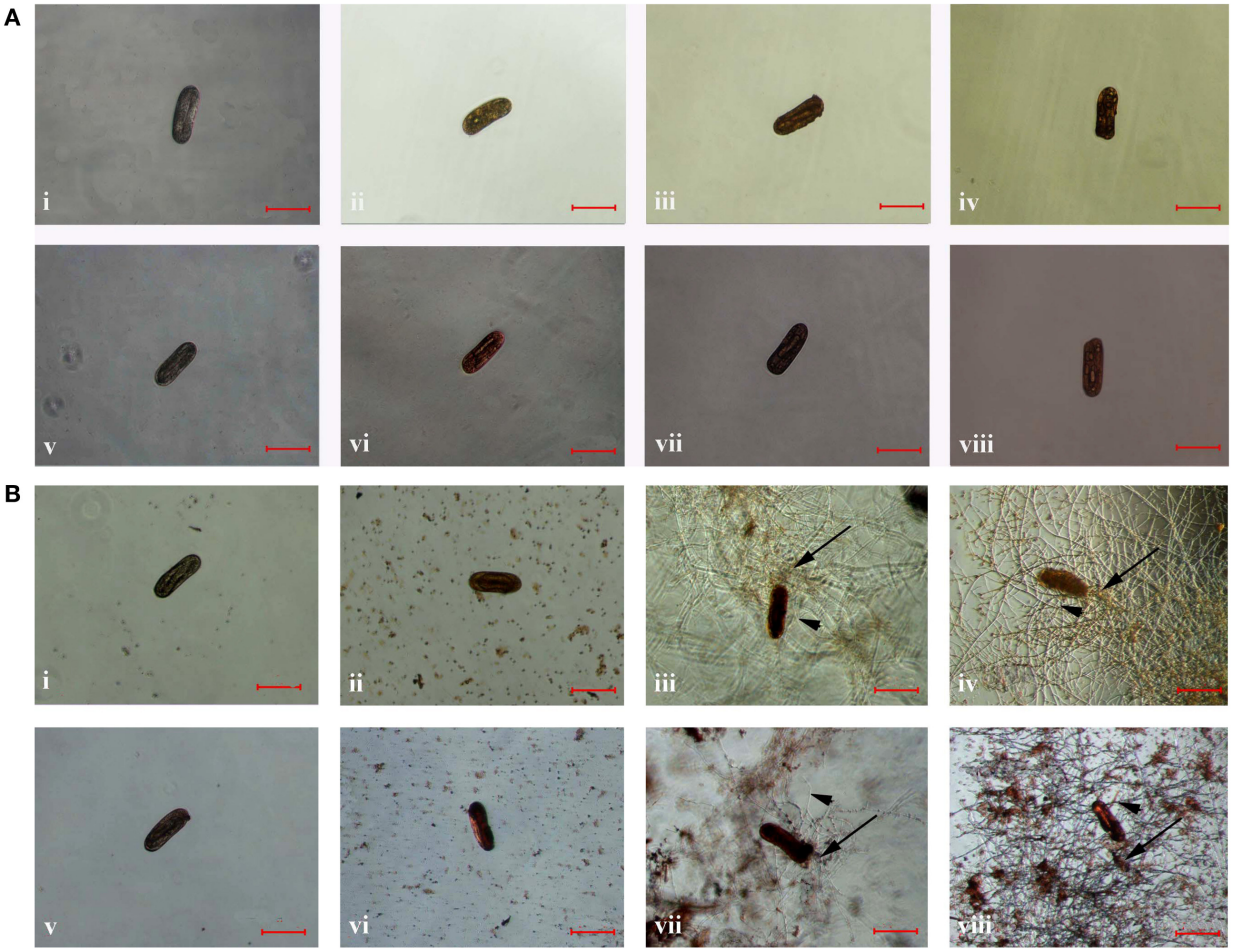
The morphological characteristics of the eggs of *H. avenae* were stained by different dyes *in vitro*. Where **(A)** the eggs were frozen in ultra-low temperature freezer (−80°C); **(i,v)** represent the eggs were not frozen but stained by AO and NR, respectively; **(ii–iv)** represent the eggs were frozen and stained by AO, and **(vi–viii)** represent the eggs were frozen and stained by NR; **(B)** the eggs were treated with 1.5 × 10^7^ conidia ml^−1^ of TL6; **(i,v)** represent the eggs were not treated with TL6 but stained by AO and NR, respectively; **(ii–iv)** represent the eggs were treated with TL6 and stained by AO, and **(vi–viii)** represent the eggs were treated with TL6 and stained by NR. Bars are in the unit of 100 μm in all the cases. The arrows name is detailed in the footnote of Figure 1.

Compared to that of the controls, the color of frozen and parasitized nematodes was significantly different after stained with the dye of AO or NR, respectively. After stained for 15 min, the color of frozen eggs changed to orange (Figures 3Aii,iii,iv) or red (Figures 3Avi,vii,viii), while the color of eggs in control group did not change significantly after stained with AO (Figure 3Ai) or NR (Figure 3Av) for 15 min. Meanwhile, the eggs color changed to orange or red after infected with the conidia suspension of TL6 and stained with AO or NR for 15 min. Especially, the color of stained eggs changed significantly when infected with the conidia (Figures 3Bii,vi) or the mycelium (Figures 3Biii,iv,vii,viii) of TL6, but the color of eggs in control group did not change (Figures 3Bi,v).

**Figure 4:**
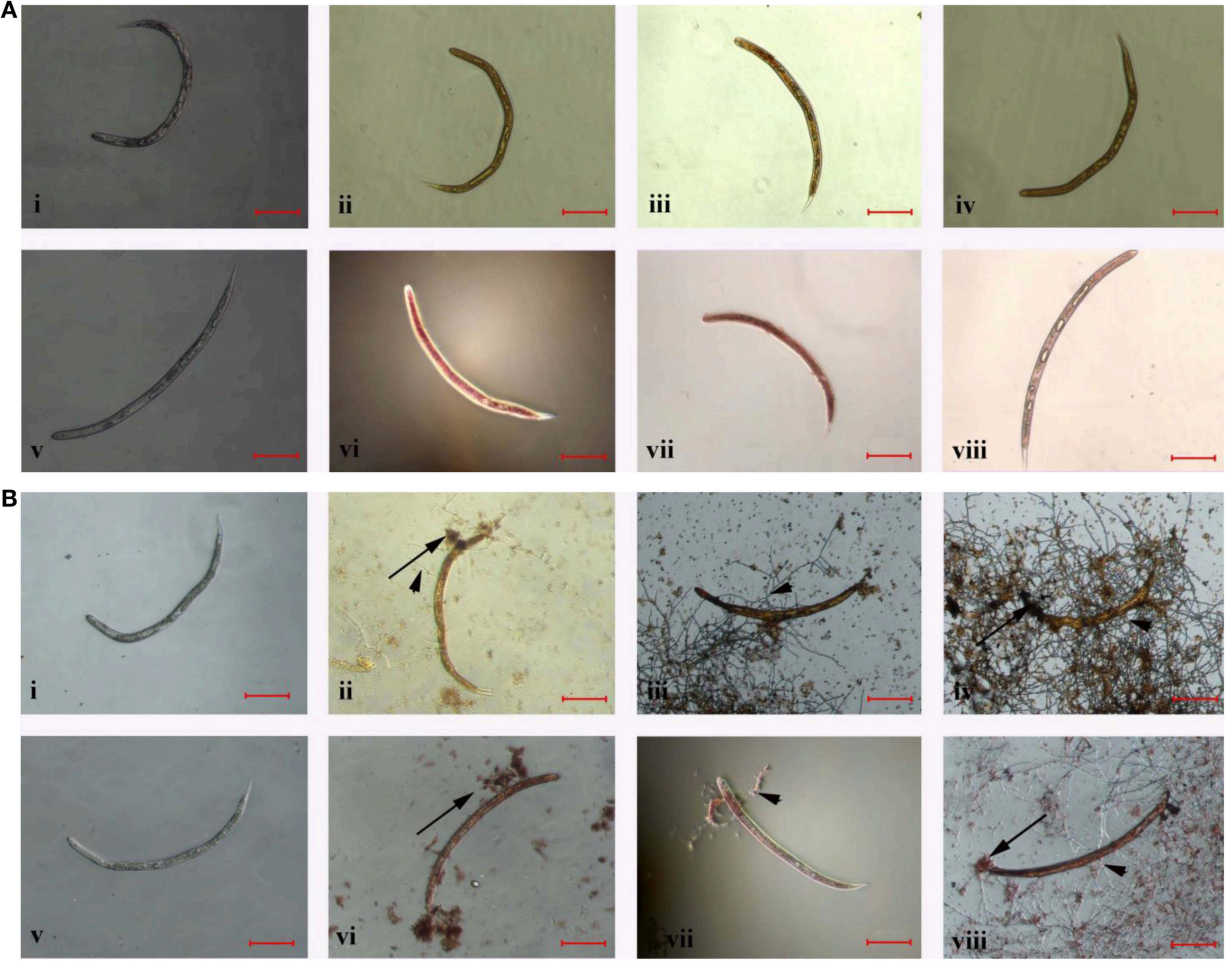
The morphological characteristics of the J2s of *H. avenae* were stained by different dyes *in vitro*. Where **(A)** the J2s were frozen in ultra-low temperature freezer (−80°C); **(i,v)** represent the J2s were not frozen but stained by AO and NR, respectively; **(ii–iv)** represent the J2s were frozen and stained by AO, and **(vi–viii)** represent the J2s were frozen and stained by NR; **(B)** the J2s were treated with 1.5 × 10^7^ conidia ml^−1^ of TL6; **(i,v)** represent the J2s were not treated with TL6 but stained by AO and NR, respectively; **(ii–iv)** represent the J2s were treated with TL6 and stained by AO, and **(vi–viii)** represent the J2s were treated with TL6 and stained by NR. Bars are in the unit of 100 μm in all the cases. The arrows name is detailed in the footnote of Figure 1.

The color of frozen J2s changed to orange (Figures 4Aii,iii,iv) or red (Figures 4Avi,vii,viii) after stained with AO or NR, but the color of stained J2s in control group did not change (Figures 4Ai,v). However, the color of stained J2s significantly changed after treated or parasitized with the conidia suspension of TL6. Especially, the color changed quickly and significantly when the conidia (Figures 4Bii,vi) or the mycelium (Figures 4Biii,iv,vii,viii) surrounded and parasitized on the surface of some J2s. In contrast, the color in the control did not change (Figures 4Bi,v).

### The symptoms of wheat seedling inoculated with eggs of *H. avenae* and different concentrations of TL6 in greenhouse experiments

**Figure 5:**
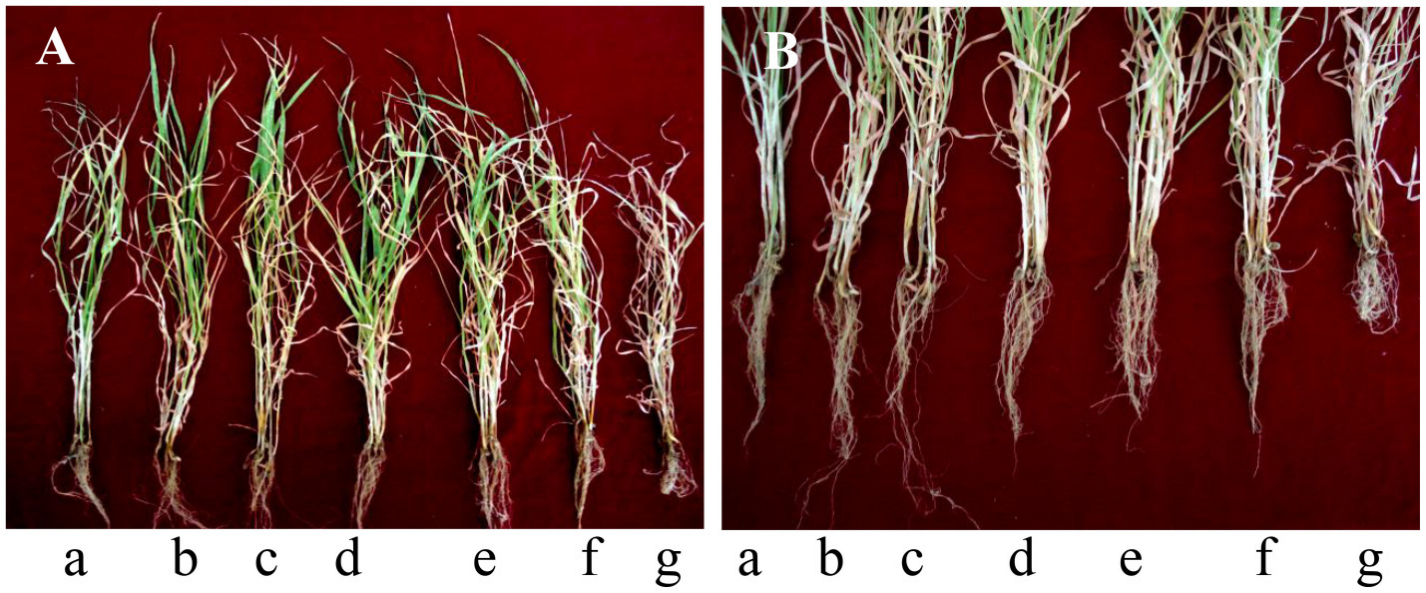
The symptoms of wheat seedlings **(A)** leaves and **(B)** roots after inoculated with eggs of *H. avenae* and different concentrations of TL6 in greenhouse experiments. **(a–g)** represent the treatments of mock-inoculated (seedlings neither inoculated with eggs nor with TL6), 1.5 × 10^7^, 1.5 × 10^6^, 7.5 × 10^5^, 3.0 × 10^5^, 1.5 × 10^5^ conidia ml^−1^ and control (seedlings inoculated with eggs but not with TL6), respectively.

Sixty-five Days after sowing, an average of 89.7% of wheat seedlings leaves showed etiolation, stunted and turned yellow after inoculated with the eggs of *H. avenae*, and even averagely 82.6% of leaves wilt readily (Figure 5Ag) compared with mock-inoculated wheat seedlings (Figure 5Aa). However, the wheat seedlings inoculated with eggs of *H. avenae* grew normally when treated with high concentrations (1.5 × 10^7^, 1.5 × 10^6^, 7.5 × 10^5^, and 3.0 × 10^5^ conidia ml^−1^) of TL6 (Figures 5Ab–e). For low concentration of treatment (1.5 × 10^5^ conidia ml^−1^), an average of 23.3% of wheat seedlings leaves showed the symptoms of slight etiolation (Figure 5Af) compared to the control (inoculated with eggs but not with TL6; Figure 5Ag).

Abnormal symptoms of roots included decreased number of roots, highly branched and hyperplasia short lateral roots, and disorder and entanglement of root system after inoculated with eggs of *H. avenae* (Figure 5Bg). In contrast, these were not observed in mock-inoculated plants (Figure 5Ba). Moreover, the eggs of *H. avenae* infected roots of wheat seedlings grew normally while treated with the high concentrations (1.5 × 10^7^, 1.5 × 10^6^, and 7.5 × 10^5^ conidia ml^−1^) of TL6 (Figures 5Bb–d). For low concentrations of treatments (3.0 × 10^5^ and 1.5 × 10^5^ conidia ml^−1^), an average of 26.5% of wheat seedlings roots appeared slightly decreased in each treatment (Figures 5Be,f), but the symptoms of roots have not appeared highly branched and hyperplasia compared to the control (inoculated with eggs of *H. avenae* but not with TL6; Figure 5Bg).

### Effect of different concentrations of TL6 on wheat seeding growth and *H. avenae* numbers in potting soil and roots

**Figure 6:**
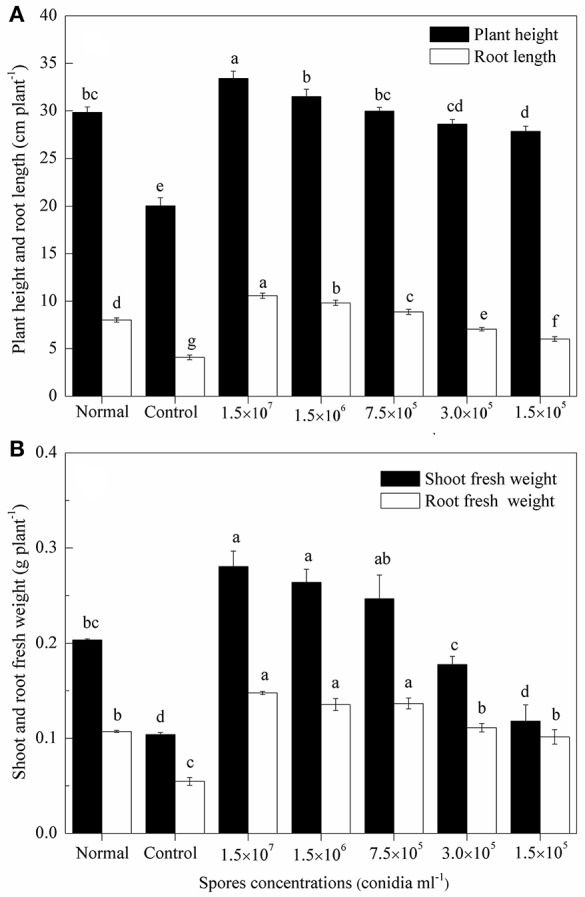
Effects of different concentrations of TL6 on wheat seedlings **(A)** height and root length, **(B)** shoot and root fresh weights after inoculated with the eggs of *H. avenae* in greenhouse experiments. Data are reported as means of replicates. Values in columns followed by different letters are significantly different at *P* < 0.05 based on Fisher's LSD test. Small bars represent the standard errors of the means. Normal (mock-inoculated) represents seedlings neither inoculated with eggs nor with TL6. Control represents seedlings inoculated with eggs but not with TL6.

Sixty-five Days after sowing, the plant height, root length and fresh weight of wheat seedlings decreased after inoculated with the eggs of *H. avenae* compared to mock-inoculated plants (*P* < 0.01), while application of different concentrations of TL6 (1.5 × 10^7^, 1.5 × 10^6^, 7.5 × 10^5^, 3.0 × 10^5^, and 1.5 × 10^5^ conidia ml^−1^) significantly increased the growth of wheat seedlings, and the effect was more pronounced with the increased concentrations of TL6, compared to the control. The relative growth rates of wheat seedling increased significantly when treated with TL6 at 1.5 × 10^7^ conidia ml^−1^, and the plant height, root length, shoot and root fresh weight increased by 66.7, 159.3 (Figure 6A), 169.1, and 170.2% (Figure 6B), respectively, compared to the control (*P* < 0.01). Meanwhile, compared to the mock-inoculated plants, treatments with the concentrations lower than 7.5 × 10^5^ conidia ml^−1^ had no significant effect on the growth of wheat plants, but had a positive effect on their growth in comparison to the control (Figure 6).

Sixty-five Days after sowing, the number of nematodes in the soil and roots of wheat seedling decreased after the combined application of different concentrations of TL6 and the eggs of *H. avenae*. Among the five concentrations of TL6, the highest concentration of 1.5 × 10^7^ conidia ml^−1^ significantly decreased the number of cysts (89.8%) and juveniles (92.7%) of *H. avenae* in soil (*P* < 0.01), and the number of juveniles (88.3%), and females (91.3%) in roots after the wheat seedlings inoculated with eggs of *H. avenae*, compared to the control (*P* < 0.01) (Table 5).

**Table 5:** Effects of different concentrations of TL6 on the populations of nematodes in wheat seeding rhizosphere after inoculated with the eggs of *H. avenae* in greenhouse experiments.

**Concentrations (conidia ml^−1^)**	**Number of cysts in soil (per 200 g of soil)**	**Number of juveniles in soil (per 20 g of soil)**	**Number of juveniles in root (per 2 g of fresh root)**	**Number of females in root (per 2 g of fresh root)**
Mock-inoculated	0.0 ± 0.0e	0.0 ± 0.0f	0.0 ± 0.0f	0.0 ± 0.0f
1.5 × 10^7^	12.7 ± 2.0d	12.2 ± 1.4e	11.0 ± 1.7e	5.2 ± 0.3e
1.5 × 10^6^	16.0 ± 2.9d	15.8 ± 1.7e	22.3 ± 2.4d	10.3 ± 0.6d
7.5 × 10^5^	24.3 ± 2.7cd	34.3 ± 2.1d	40.7 ± 3.2c	14.8 ± 1.1c
3.0 × 10^5^	31.7 ± 3.9c	68.8 ± 5.0c	45.7 ± 3.8c	17.7 ± 0.8bc
1.5 × 10^5^	56.3 ± 4.1b	111.9 ± 6.2b	68.7 ± 3.3b	19.2 ± 1.7b
Control	124.7 ± 7.4a	166.3 ± 6.3a	94.0 ± 4.0a	59.5 ± 1.7a
Mean square	5,321.94	11,280.05	3,261.33	1,520.31
*F* value	115.81	237.11	126.60	340.19
*P* value	0.0001	0.0001	0.0001	0.0001

*Mean ± SE in a column followed by different letters are significantly different at P < 0.05, based on Fisher's LSD test. Mock-inoculated represents seedlings neither inoculated with eggs nor with TL6. Control represents seedlings inoculated with eggs but not with TL6*.

### Determination of chitinase and β-1, 3-glucanase activity

Compared to the mock-inoculated treatment, the activity of chitinase and β-1, 3-glucanase in wheat seedling roots were increased significantly after inoculation with the eggs of *H. avenae* (*P* < 0.01). However, a higher increase in chitinase and β-1, 3-glucanase activity was observed after the wheat seedlings treated with different concentrations of TL6 (1.5 × 10^7^, 1.5 × 10^6^, 7.5 × 10^5^, 3.0 × 10^5^, and 1.5 × 10^5^ conidia ml^−1^) and eggs of *H. avenae*. The activity of chitinase and β-1, 3-glucanase reached its maximum at the 20^th^ and 15^th^ Days after inoculation with TL6 and thereafter it declined. In the wheat seedlings roots, the maximum activity of chitinase and β-1, 3-glucanase increased from 0.4 to 43.9% (Table 6), and 6.1 to 32.0% (Table 7) for the wheat seedlings inoculated with the different concentrations of TL6 and eggs at the 20^th^ and 15^th^ Days, respectively, compared to the control. Specifically, the chitinase and β-1, 3-glucanase activity significantly increased by 43.9 and 32.0% in wheat seedlings roots after the combined inoculation with the eggs and highest concentrations of TL6 at 1.5 × 10^7^ conidia ml^−1^, compared to the control (*P* < 0.01).

**Table 6:** Effects of different concentrations of TL6 on the activity of chitinase in wheat seedling roots after inoculated with the eggs of *H. avenae* in greenhouse experiments.

**Concentrations (conidia ml^−1^)**	**Days after inoculation with TL6 (d)**					
	**5**	**10**	**15**	**20**	**30**	**40**
	**Chitinase activity (nmol mg^−1^ protein min^−1^)**					
Mock-inoculated	13.87 ± 0.12 c	14.59 ± 0.68 e	16.63 ± 0.30 e	18.24 ± 0.47f	15.87 ± 0.11f	14.71 ± 0.32d
1.5 × 10^7^	19.55 ± 0.54a	24.54 ± 0.59a	26.39 ± 0.62a	27.34 ± 0.53a	24.45 ± 0.23a	21.65 ± 0.81a
1.5 × 10^6^	19.36 ± 0.58a	22.31 ± 0.53b	24.67 ± 0.62b	25.45 ± 0.52b	21.43 ± 0.86b	20.82 ± 0.58a
7.5 × 10^5^	18.44 ± 0.53a	19.47 ± 0.23c	21.29 ± 0.77c	22.58 ± 0.29c	20.13 ± 0.53bc	18.10 ± 0.44b
3.0 × 10^5^	16.38 ± 0.30b	19.27 ± 0.12c	20.55 ± 0.16c	20.97 ± 0.58d	18.93 ± 0.60cd	18.17 ± 0.25b
1.5 × 10^5^	15.59 ± 0.83b	17.29 ± 0.23d	18.62 ± 0.34d	19.08 ± 0.17e	17.64 ± 0.24de	16.06 ± 0.42c
Control	15.36 ± 0.36bc	16.70 ± 0.64d	18.34 ± 0.40d	19.00 ± 0.58e	16.54 ± 0.57ef	15.83 ± 0.35c
Mean square	14.52	34.73	37.33	36.60	27.02	20.28
*F* value	18.48	49.55	49.77	55.17	34.26	28.55
*P* value	0.0001	0.0001	0.0001	0.0001	0.0001	0.0001

*Mean ± SE in a column followed by different letters are significantly different at P < 0.05, based on Fisher's LSD test. Mock-inoculated represents seedlings neither inoculated with eggs nor with TL6. Control represents seedlings inoculated with eggs but not with TL6*.

**Table 7:** Effects of different concentrations of TL6 on the activity of β-1, 3-glucanase in wheat seedling roots after inoculated with the eggs of *H. avenae* in greenhouse experiments.

**Concentrations (conidia ml^−1^)**	**Days after inoculation with TL6 (d)**					
	**5**	**10**	**15**	**20**	**30**	**40**
	**β-1, 3-glucanase activity (nmol mg**^**−1**^ **protein min**^**−1**^**)**					
Mock-inoculated	9.44 ± 0.28e	11.96 ± 0.57d	13.67 ± 0.23e	12.40 ± 0.23e	11.23 ± 0.09d	10.29 ± 0.39e
1.5 × 10^7^	16.34 ± 0.18a	17.69 ± 0.58a	19.78 ± 0.19a	18.63 ± 0.49a	17.28 ± 0.38a	17.36 ± 0.33a
1.5 × 10^6^	14.11 ± 0.01b	16.34 ± 0.40b	18.98 ± 0.40a	17.53 ± 0.31b	16.73 ± 0.38a	16.52 ± 0.52b
7.5 × 10^5^	13.22 ± 0.51bc	16.38 ± 0.21b	17.49 ± 0.35b	17.50 ± 0.21b	16.98 ± 0.17a	16.74 ± 0.10b
3.0 × 10^5^	12.68 ± 0.59cd	15.89 ± 0.36b	16.38 ± 0.46c	15.39 ± 0.18c	14.55 ± 0.45b	13.67 ± 0.65c
1.5 × 10^5^	11.98 ± 0.10d	14.07 ± 0.41c	15.89 ± 0.34cd	15.08 ± 0.14cd	14.32 ± 0.22b	13.21 ± 0.13cd
Control	11.78 ± 0.51d	13.58 ± 0.29c	14.98 ± 0.46d	14.41 ± 0.22d	13.22 ± 0.32c	12.25 ± 0.46d
Mean square	13.74	11.88	14.09	14.00	15.04	21.03
*F* value	32.69	22.17	36.14	61.25	51.19	40.75
*P value*	0.0001	0.0001	0.0001	0.0001	0.0001	0.0001

*Mean ± SE in a column followed by different letters are significantly different at P < 0.05, based on Fisher's LSD test. Mock-inoculated represents seedlings neither inoculated with eggs nor with TL6. Control represents seedlings inoculated with eggs but not with TL6*.

### Measurement of total flavonoids and lignin contents

Total flavonoids in the roots of wheat seedlings increased significantly as results of application of different concentrations of TL6 (1.5 × 10^7^, 1.5 × 10^6^, 7.5 × 10^5^, 3.0 × 10^5^, and 1.5 × 10^5^ conidia ml^−1^) and inoculation with eggs of *H. avenae* in comparison to the mock-inoculated plants. Specially, application of higher concentrations of TL6 presented higher total flavonoids in the roots of wheat seedlings, but the lowest concentration increased the total flavonoids contents slightly, compared to the control. The maximum content of total flavonoids in wheat seedling roots was recorded at the 30^th^ Day after application of different concentrations of TL6 and inoculation with eggs of *H. avenae* (*P* < 0.01). Moreover, the total flavonoids in the roots of wheat seedlings increased after inoculation with the eggs of *H. avenae* alone, compared to that of mock-inoculated plants (Table 8).

**Table 8:** Effects of different concentrations of TL6 on the contents of total flavonoids in wheat seedling roots after inoculated with the eggs of *H. avenae* in greenhouse experiments.

**Concentrations (conidia ml^−1^)**	**Days after inoculation with TL6 (d)**					
	**5**	**10**	**15**	**20**	**30**	**40**
	**Total flavonoids (mg g**^**−1**^ **FW)**					
Mock-inoculated	1.58 ± 0.01d	1.85 ± 0.04e	2.06 ± 0.05e	2.15 ± 0.07e	2.31 ± 0.10e	2.26 ± 0.10d
1.5 × 10^7^	2.28 ± 0.03a	2.54 ± 0.08a	2.78 ± 0.06a	2.95 ± 0.08a	3.19 ± 0.10a	3.05 ± 0.10a
1.5 × 10^6^	2.21 ± 0.06a	2.37 ± 0.05b	2.51 ± 0.05b	2.80 ± 0.06ab	3.02 ± 0.09ab	2.93 ± 0.12a
7.5 × 10^5^	2.17 ± 0.06ab	2.28 ± 0.06bc	2.39 ± 0.06bc	2.65 ± 0.05bc	3.04 ± 0.09ab	2.77 ± 0.14b
3.0 × 10^5^	2.06 ± 0.03b	2.17 ± 0.04cd	2.27 ± 0.07cd	2.43 ± 0.11cd	2.80 ± 0.07bc	2.79 ± 0.10b
1.5 × 10^5^	1.86 ± 0.03c	2.05 ± 0.07d	2.13 ± 0.05de	2.34 ± 0.09de	2.63 ± 0.07cd	2.45 ± 0.08c
Control	1.76 ± 0.04c	2.03 ± 0.05d	2.12 ± 0.03de	2.23 ± 0.05de	2.48 ± 0.04de	2.41 ± 0.12c
Mean square	0.20	0.16	0.20	0.27	0.32	0.26
*F* value	38.06	16.81	23.38	16.04	16.00	6.75
*P value*	0.0001	0.0001	0.0001	0.0001	0.0001	0.0001

*Mean ± SE in a column followed by different letters are significantly different at P < 0.05, based on Fisher's LSD test. Mock-inoculated represents seedlings neither inoculated with eggs nor with TL6. Control represents seedlings inoculated with eggs but not with TL6. FW represents fresh weights*.

The contents of lignin in wheat seedling roots increased after inoculated with the eggs of *H. avenae* alone, while application of different concentrations of TL6 significantly increased the lignin contents in wheat seedling roots, compared to the mock-inoculated wheat seedlings (*P* < 0.01). There was a trend that the content of lignin in wheat seedling roots increased consistently with the increase of TL6 concentration or incubation days. The highest increase in the contents of lignin was achieved after application of TL6 at 1.5 × 10^7^ and 1.5 × 10^6^ conidia ml^−1^, followed by 7.5 × 10^5^, 3.0 × 10^5^, and 1.5 × 10^5^ conidia ml^−1^, respectively. Compared to the control, the highest increase in the contents of lignin by 34.9% and 32.2% at the 40^th^ Day after inoculated with eggs and TL6 at the concentrations of 1.5 × 10^7^ and 1.5 × 10^6^ conidia ml^−1^, respectively (Table 9).

**Table 9:** Effects of different concentrations of TL6 on the contents of lignin in wheat seedling roots after inoculated with the eggs of *H. avenae* in greenhouse experiments.

**Concentrations (conidia ml^−1^)**	**Days after inoculation with TL6 (d)**					
	**5**	**10**	**15**	**20**	**30**	**40**
	**Lignin content (mg g**^**−1**^ **DW)**					
Mock-inoculated	0.85 ± 0.03d	0.93 ± 0.04e	0.96 ± 0.03f	0.99 ± 0.05d	1.03 ± 0.04d	1.04 ± 0.04d
1.5 × 10^7^	1.39 ± 0.05a	1.51 ± 0.06a	1.74 ± 0.05a	1.91 ± 0.08a	1.92 ± 0.07a	1.97 ± 0.08a
1.5 × 10^6^	1.39 ± 0.04a	1.48 ± 0.02ab	1.66 ± 0.07ab	1.89 ± 0.05ab	1.94 ± 0.04a	1.93 ± 0.11a
7.5 × 10^5^	1.30 ± 0.03a	1.36 ± 0.03bc	1.58 ± 0.04bc	1.71 ± 0.09b	1.73 ± 0.08b	1.75 ± 0.10ab
3.0 × 10^5^	1.18 ± 0.03b	1.29 ± 0.05cd	1.48 ± 0.04cd	1.52 ± 0.02c	1.52 ± 0.05c	1.58 ± 0.07bc
1.5 × 10^5^	1.17 ± 0.05b	1.28 ± 0.02cd	1.41 ± 0.06de	1.44 ± 0.02c	1.48 ± 0.06c	1.51 ± 0.04c
Control	1.03 ± 0.04c	1.16 ± 0.07d	1.28 ± 0.04e	1.34 ± 0.04c	1.37 ± 0.05c	1.46 ± 0.05c
Mean square	0.11	0.12	0.21	0.31	0.31	0.30
*F* value	25.80	19.05	27.92	31.81	31.27	19.12
*P* value	0.0001	0.0001	0.0001	0.0001	0.0001	0.0001

*Mean ± SE in a column followed by different letters are significantly different at P < 0.05, based on Fisher's LSD test. Mock-inoculated represents seedlings neither inoculated with eggs nor with TL6. Control represents seedlings inoculated with eggs but not with TL6. DW represents dry weights*.

## Discussion

Previous studies report that the nematode of *H. avenae*, a root pathogen of cereals, is found in more than 31 wheat growing countries causing significant economic yield losses, particularly in those countries where rainfed cereal systems predominate (Nicol et al., 2003). Our previous studies have demonstrated that *T. longibrachiatum* is a well-known bio-control agent against several plant pathogens, which including the fungi and *H. avenae* cysts without hazardous effects to the environment (Zhang et al., 2014a,b), but little information is available regarding the process of the conidia suspension of TL6 infecting the same eggs or J2s at different time points, and also a rapid method to assay the viability of *H. avenae in vitro*. To our knowledge, this study is the first to discover the detailed process of the conidia suspension of TL6 infecting the same eggs or J2s in each day of the observation by microscope, and also find the vital dyes of AO and NR were considered as the rapid method to assay the viability of *H. avenae*. Meanwhile, the possible mechanisms for the potential of TL6 as a bio-control agent against the eggs and J2s of *H. avenae* were assessed in both greenhouse experiments and *in vitro* tests. Interestingly, the consistent results from the two assessment facilities indicate that *T. longibrachiatum* T6 (TL6) has the potential to be used as an effective bio-control agent in suppressing of *H. avenae*, and the possible mechanisms are due to the direct parasitic and lethal effect of TL6 on the eggs and J2s activity, and the induced defense response (accumulation of defense compounds and up-regulation of enzymes) in wheat plants.

*In vitro* studies showed that the conidia suspension of TL6 had strong inhibitory and parasitic effects on the eggs of *H. avenae* and J2s. Moreover, the parasitic effect of the conidia suspension of TL6 on eggs of *H. avenae* was faster than the effect on their cysts of *H. avenae* and the J2s of *M. incognita*, compared with the previous studies (Zhang et al., 2014b, 2015). Also, the lethal effect on the J2s was faster than the inhibitory effect on the eggs in our present study. To study the potential of *Trichoderma* species in controlling of nematodes, Yang et al. (2010) evaluated the fungal filtrates of 329 *Trichoderma* strains against *Panagrellus redivivus* and *Caenorhabditis elegans*, and found lower nematicidal effect of the strain of *T. longibrachiatum* against either nematode. This may be related to the role of mutual recognition of the metabolites (an active compound of acetic acid) produced by the conidia suspension of TL6 and the chemicals contained by eggs shell and the body wall of nematodes (Djian et al., 1991). Previous study show that parasitism is one of the modes of action of *Trichoderma* species against *M. javanica*, but the percentages of parasitism on the eggs and J2s of *M. javanica* were very low (Sharon et al., 2009). In our present study, we found the percentages of parasitism on the eggs and J2s of *H. avenae* were higher than the parasitic effect on the eggs and J2s of *M. javanica in vitro*, compared with the previous study. It may be related to the contact opportunity of the conidia suspension of TL6 with the eggs shell and the parasites, or the species of *Trichoderma* strains (Sharon et al., 2009). Moreover, previous studies revealed that *P. lilacinus* was an opportunistic fungus, and it usually parasitizes on nematode eggs and the percent parasitism was related to the length of time when it contacts with eggs (Leij De et al., 1992; Bonants et al., 1995; Oclarit and Cumagun, 2009). Similarly, *P. chlamydosporia* usually infects the eggs and females, and the ability to colonize different nematodes varies greatly (Bourne et al., 1996). However, the findings from the present study revealed that the strain of TL6 not only parasitized or colonized on the eggs, but also parasitized the J2s of *H. avenae*.

Previous studies have demonstrated that some nematophagous fungi, such as the egg-parasitic fungi *Paecilomyces* spp. (Huang et al., 2004) and *Pochonia* spp. (Tikhonov et al., 2002), have the ability to trap nematodes, infect nematode eggs, and suppress the hatching of juveniles, thereby reducing nematodes population. In the present study, our results showed that the inhibitory effect of TL6 (1.5 × 10^5^ spores ml^−1^) on the hatching of eggs was stronger than the inhibitory effect of *P. lilacinus* and *P. chlamydosporia* on the hatching of individual eggs (41.3 and 36.0%) of *Meloidogyne* spp. at 1.0 × 10^5^ spores ml^−1^ (Sun et al., 2006). Such differences may be related to the strains, the mechanism of infection and the host of bio-control fungi (Stirling, 1991; Kerry, 1997; Jeffries et al., 2003; Tchabi et al., 2010). Our results also revealed that the highest parasitic and lethal effect of *F. oxysporum* on the eggs and J2s of *H. avenae* were lower than 37.0%, and significantly lower than the parasitic and lethal effect of TL6. In a similar study, Yan et al. (2011) demonstrated that the average control efficacy of five isolates of *Fusarium* sp. on *M. incognita* was 35.7%.

To our knowledge, this study is the first in the scientific literature to report and document the detailed process of the conidia suspension of TL6 infecting the same *H. avenae* eggs or J2s in each day of the observation by microscope, and the direct parasitism was probably the important mode of action of TL6 in suppressing of *H. avenae* eggs and J2s growth and development. Zhang et al. (2014b) have been reported that the process of *H. avenae* cysts infestation by *T. longibrachiatum in vitro*, but there are no related information regard to the process of TL6 infecting *H. avenae* eggs or J2s, especially the process of TL6 infecting the same eggs or J2s of *H. avenae* at different days. Also, our results indicate that TL6 may produce the metabolites and enzymes that cause the physiological disorder of eggs, dissolve nematodes body, and severely affect their physical vitality (Khambay et al., 2000; Sharon et al., 2007; Gortari and Hours, 2008; Pau et al., 2012). However, this needs to be further investigated. Moreover, similar results were reported that the fermentation broth of *P. lilacinus* was found to contain acetic acid that inhibited the eggs growth (Djian et al., 1991). The parasitism and inhibition of cysts through the increased extracellular chitinase activity serve as the main mechanism with which the fermentation broth of *T. longibrachiatum* against *H. avenae* cysts (Zhang et al., 2014b).

Some species of insecticides and nematicides may kill the nematodes eggs, but eggs death cannot be determined unless the contents are visibly damaged (Donald and Niblack, 1994), and also some fungal biological control agents in eggs can be detected only after mycelium has proliferated within the eggs (Zetsche and Meysman, 2012). Moreover, it remains a challenge to count live and dead organisms in a concentrated sample of debris in recent years (Fischel, 1908). Staining procedures have been used for over 100 years to differentiate living cells from dead cells (Crippen and Perrier, 1974). Vital stains acridine orange and tetrazolium red for differentiating viable and nonviable eggs of *H. glycines* provide a useful tool for laboratory and greenhouse tests (Donald and Niblack, 1994). Less toxic vital stain of neutral red has been successfully applied to copepods (Elliott and Tang, 2009), and recently, the applicability to natural field assemblages of zooplankton has been further promoted (Yan et al., 2011). For the first time in the literature, our study determined the details of the viability of eggs and J2s of *H. avenae* after treated with biological control strain of TL6 or under ultra-low temperature conditions (−80°C). We showed that the vital dyes of AO and NR are rapid, effective, and efficient in the assay of the viability of *H. avenae*.

Our earlier study showed that the fermentation broth of *T. longibrachiatum* applied at appropriate concentrations suppressed *H. avenae* cysts development and infection, and at the same time promoted the growth of wheat that was inoculated with the cysts of *H. avenae* and *T. longibrachiatum* (Zhang et al., 2014a). Leij De and Kerry (1993) reported that the number of root-knot nematodes in soil was reduced by 90% after application of *P. chlamydosporia*. An interesting finding from the present study revealed that a high efficiency of TL6 in suppressing of *H. avenae* development and infection, and promoting wheat seedling growth in comparison to the previous studies (Leij De and Kerry, 1993; Zhang et al., 2014a). Meanwhile, an added value from the present study was that in the study of nematodes, eggs can be used as the inoculum nematodes.

Furthermore, a number of studies have been reported on microorganisms mediated induction of resistance in different plant species (Lopez and Hernández, 2014). However, there are only a few reports on the application of plant growth promoting microorganisms for the induction of resistance in wheat against *H. avenae*. Especially there are no reports about the colonization of wheat roots by the strain of TL6 inducing the activity of chitinase and β-1, 3-glucanase, and increasing the content of total flavonoids and lignin resistance to *H. avenae* infection. At our knowledge, the current study is firstly revealed that wheat inoculated with antagonistic bio-control strain of TL6 under *H. avenae* infection condition induces resistance enzymes (chitinase and β-1, 3-glucanase) and defense compounds (total flavonoids and lignin) responsible for a reprogrammed metabolic cascade related to plant defense and signaling. Our results also provide an insight into the possibilities of applying the bio-control strain of TL6 for managing *H. avenae* disease (Harman et al., 2004; Bakker et al., 2007; Singh et al., 2014), which would be eco-friendly means to manage *H. avenae* disease and contributes to the maintenance of plant and soil health.

Chitinase and β-1, 3-glucanase are the two important pathogenesis-related (PR) proteins in plant tissue and the accumulation of these can contribute to plant defense response against the pathogen infection (Kauffmann et al., 1987; Poonam et al., 2013). We discovered that the activity of chitinase and β -1, 3-glucanase significantly increased when the wheat seedlings were inoculated with the strain of TL6 compared to un-inoculated plants, and that the high concentrations of TL6 remarkably promoted the chitinase and β-1, 3-glucanase activities. The maximum activities of chitinase and β-1, 3-glucanase were recorded at the 20^th^ and 15^th^ Days after the combined application of different concentrations of TL6 and eggs in soil. Guzmán-Valle et al. (2014) also revealed that the activity of glucanase, chitinase and peroxidase in bulbs and roots of different varieties of onion (*Allium cepa* L.) were increased after application of *T. asperellum* and *Sclerotium rolfsii*. Our results were in the line of previous studies which demonstrate that the interaction between *Trichoderma* species and plant roots induces the increase in enzyme activity and that the magnitude of the activity is related to the infection of soil-borne plant pathogens (Harman, 2006; Salas-Marina et al., 2011; Singh et al., 2013). Moreover, a number of previous studies have demonstrated that root colonization by *T. harzianum* strains has been shown to increase the level of resistance-related enzymes (Howell et al., 2000; Evans et al., 2003), and especially root colonization by *T. longibrachiatum* significantly increased the specific activities of resistance-related enzymes (peroxidase, polyphenol oxidase and phenylalanine ammonia lyase) to induce the resistance of wheat against *H. avenae* infection (Zhang et al., 2014a). Singh et al. (2016) reported that wheat seedlings co-inoculated with *Bacillus amyloliquefaciens* B-16 and *T. harzianum* UBSTH-501 increased the activity of phenylalanine ammonia lyase, peroxidase, chitinase, β-1, 3-glucanase and other enzymes related to induce systemic resistance responses against *Bipolaris sorokiniana*. Unlike previous studies, our present study discovered the resistance-related enzymes (chitinase and β -1, 3-glucanase) increased in wheat after inoculated with the strain of TL6 and *H. avenae* eggs, which may serve as the main mechanism responsible for TL6 against *H. avenae*.

Flavonoids are an important group of secondary metabolites in plants that can function as an “inducer” that induces the resistance of plants against pathogen infections (Bahraminejad et al., 2008). Lignin is a complex polymer of hydroxylated and methoxylated phenylpropane units, and also cell wall lignifications play a significant role in the incorporation of lignin into plant cell wall so that improving plant resistance to the pathogen invasion. In the present study, we discovered the eggs of *H. avenae* infection significantly induced the contents of total flavonoids and lignin in the roots of wheat seedlings compared to the un-inoculated plants, and the most notable increase in the contents of total flavonoids and lignin occurred after the combined application of different concentrations of TL6 and eggs in soil at different stages. The maximum contents of total flavonoids and lignin were recorded at the 30^th^ and 40^th^ Days after the combined application of different concentrations of TL6 and eggs in soil. In studying faba bean (*Vicia faba* L.), Abd El-Rahman and Mohamed (2014) found that the application of benzothiadiazole and *T. harzianum* combination increased total flavonoids (3.1 and 2.9-fold) and lignin content (81.3% and 59.5%) when the faba bean was infected by the pathogens of *Botrytis faba* or *B. cinerea* in comparison to the untreated control. Moreover, previous studies demonstrated that the induction of host plants with resistance by *Trichoderma* spp. has been described to be associated with activation of disease resistance mechanisms and production of a wide range defense compounds mainly including peroxidase, phenylalanine ammonia lyase, flavonoids (Karthikeyan et al., 2006; Magro et al., 2009; Govindappa et al., 2010; Nianlai et al., 2010), and the accumulation of lignin and pectin in plants cell walls (Al-Hakimi and Al-Ghalibi, 2007; Nianlai et al., 2010). Our results are in agreement with these alterations in plants which may contribute to the resistance by stopping pathogen invasion or slowing down the penetration process, thus allowing the activation of further defense mechanisms in plants (Sticher et al., 1997). Meanwhile, our present results indicate that the changes of lignin contents in wheat seedlings roots may be related to resistance in incorporating of lignin into plant cell wall is bound to make it more resistance to the nematodes invasion. Also, lignified cell walls could also constitute a defense barrier preventing free nutrient movement and therefore help to starve the nematodes (Sticher et al., 1997). However, a more detailed mechanism for the induction of host plants with resistance to plant parasitic nematodes by the strain of TL6 need to be solved in the future studies.

In addition, Gupta et al. (2017) reported that a consortium of bioinoculants of *B. megaterium, T. harzianum* ThU and *Glomus intraradices* significantly induced the total phenolic and flavonoid contents in chamomile (*Matricaria recutita* L.) resistance to root-knot nematode infection. Similarly, Singh et al. (2016) found higher amounts of phenolic acids (gallic acid and ferulic acid) were accumulated in wheat leaves after co-inoculated with *B. amyloliquefaciens* B-16 and *T. harzianum* UBSTH-501, compared to the individually inoculated and un-inoculated control plants. Finally, our results indicate that roots colonization by plant growth promoting microorganism of TL6 induces the accumulation of plant defense compounds and up-regulation of enzymes to establish symbiotic interactions as well as to fight against pathogens during the pathogen infection, which in lined with the findings of Harman et al. (2004) and Singh et al. (2013), who showed that colonization of plants with the bio-agents of *T. harzianum, B. amyloliquefaciens* and other plant growth promoting microorganisms can induce the plant against pathogens.

## Conclusion

Our study suggests that the conidia suspension of TL6 has a broad prospect on the prevention and control of *H. avenae* eggs and J2s *in vitro* and greenhouse, and has the potential to be used as an effective bio-control agent for *H. avenae*. The main mechanisms of the strain of TL6 against the eggs and J2s of *H. avenae* were due to (i) the direct parasitic and lethal effect of TL6 on the activity of the eggs and J2s development, and (ii) the promoting effect on the wheat growth and development, and improving chitinase and β-1, 3-glucanase activities, and the flavonoids and lignin contents in plants resistance to *H. avenae* infection. In addition, AO and NR can be considered as the rapid vital dyes to assay the viability of *H. avenae*. Further studies are required to develop effective methodology with which active agents in nematicidal activity can be identified and isolated. Also, studies are needed to determine the functionality of biological nematicide genes in the strain of TL6 and the persistence of the strain in various life stages when interacting with nematodes.

## Author contributions

SZ and WJ designed the experiments with the help of BX. SZ and JL performed the microscopic observation of the infection process of TL6 on *H. avenae* eggs and J2s. SZ, YG, and BH performed the greenhouse experiments and analyzed the data, with the help of BX. SZ and YG wrote the manuscript, and revised and approved the final manuscript with the help of BX.

### Conflict of interest statement

The authors declare that the research was conducted in the absence of any commercial or financial relationships that could be construed as a potential conflict of interest.

## Abbreviations

AO: Acridine orange
FO: *Fusarium oxysporum*
J2s: Second stage juveniles
N-AcG: N-acetyl glucosamine
NR: Neutral red
PDA: Potato dextrose agar
TL6: *Trichoderma longibrachiatum* T6.
